# CampyTube: Seamless Integration of a Molecular Test and Lateral Flow Detection of *Campylobacter* in a Single Vial

**DOI:** 10.3390/bios15080497

**Published:** 2025-08-01

**Authors:** Natalia Sandetskaya, Andreas Kölsch, Kai Mattern, Vanessa Vater, Dirk Kuhlmeier, Florian Priller

**Affiliations:** 1 MicroDiagnostics Group, Fraunhofer Institute for Cell Therapy and Immunology IZI, Perlickstrasse 1, 04103 Leipzig, Germany; 2 Hygiena Diagnostics GmbH, Hermannswerder 17, 14473 Potsdam, Germany

**Keywords:** *Campylobacter*, LAMP, lateral flow detection, point-of-care test, integrated diagnostics

## Abstract

Background: The efficient control of hygiene and *Campylobacter*’s contamination status at various steps of poultry meat production is essential for the prevention of *Campylobacter* transmission to humans. Microbiological methods are laborious and time-consuming, and molecular methods of detection are often too skill- and infrastructure-demanding. Methods: We have developed CampyTube, a simple and user-friendly format for the integration of isothermal DNA amplification with embedded instrument-free detection on a miniaturized lateral flow test in a single vial. All test components, from the dry amplification reagents to the mini lateral flow tests, are incorporated into a standard single vial, which is closed after the addition of the liquid sample and never has to be opened again. This ensures the absolute prevention of carry-over contamination and makes the system very safe and simple to use in point-of-need settings. Results: As few as 60 *Campylobacter* genome copies per reaction could be successfully detected with CampyTube. We have primarily developed and evaluated CampyTube for the detection of *Campylobacter* in chicken neck skin samples and could reach 100% sensitivity and 100% specificity in the samples exceeding the regulatory limit of 1000 CFU/g confirmed microbiologically, while the sensitivity in all samples that tested positive using qPCR (1.4 × 10^2^–2.5 × 10^6^ genome copies/g) was 71.1%. We discuss the impact of sample preparation on CampyTube performance and suggest further options for test optimization. Conclusions: CampyTube is a highly versatile and efficient, yet simple, affordable, and material-saving system that can be adapted for other targets and sample types.

## 1. Introduction

Campylobacteriosis, an acute diarrhoeal disease in humans, remains one of the most frequent foodborne infections worldwide [1,2,3]. According to the 2019 report by the European Food Safety Authority (EFSA), a total of 220,682 human cases of Campylobacteriosis were confirmed in the European Union, with an average notification rate of 59.7 cases per 100,000 individuals [4,5]. The consumption of insufficiently thermally processed poultry meat or the contamination of cooking utensils and surfaces during meat processing are the most common origins of infection [6].

*Campylobacter jejuni* and *C. coli* account for almost 90% of the reported cases of human Campylobacteriosis [6]. However, other species, including *C. lari, C. upsaliensis, and C. concisus*, have also been identified as potential causes of infection [6,7].

*Campylobacter* is commonly found in the intestinal tracts of various wild and domestic animals, as well as birds, and is prevalent throughout the poultry production environment. Chickens serve as a natural reservoir for *Campylobacter* species and are the primary source of transmission to humans [8]. Currently, no single control method effectively eliminates *Campylobacter* contamination in the poultry industry [9]. Therefore, the strict and efficient monitoring and control of hygiene and contamination status at various steps of poultry meat production must be maintained for the prevention of *Campylobacter* transmission to humans.

Current methods for *Campylobacter* detection in meat production facilities rely on laborious and time-consuming microbiological methods [3,10]. More rapid, advanced, and sensitive methods like nucleic acid amplification, DNA hybridization and fingerprinting techniques, mass spectrometry techniques, and biosensors are being developed, yet they demand special equipment, adequate lab infrastructure, and skilled personnel [6,11,12,13,14]. A user-friendly molecular detection format suitable for various test throughputs (i.e., compact and cost-effective) could significantly facilitate hygiene control for *Campylobacter*.

We have developed CampyTube—a test format that seamlessly integrates molecular detection with an instrument-free qualitative readout. CampyTube is based on the molecular isothermal amplification of *Campylobacter* DNA with integrated visual detection on a miniaturized lateral flow test (LFT) in a single closed vial. All test components are incorporated in a standard 0.5 mL vial with a screw cap, which remains closed after the addition of the sample and never has to be opened again, thus eliminating any cross-contamination risks. These characteristics make CampyTube especially beneficial for the point-of-need settings.

We have manufactured and evaluated the CampyTube tests for the detection of *Campylobacter* spp. (further referred to as *Campylobacter*) in chicken neck skin samples. In the current manuscript, we describe the principle of the test, its advantages over the State-of-the-Art methods, and propose ideas for the further development of the CampyTube-based detection of pathogens.

While the regulatory framework currently only allows for cultural determination in accordance with EN ISO 10272-1 in Europe [10], rapid molecular tests such as CampyTube could represent a future alternative. The new test platform aims to complement hygiene monitoring with a modern rapid testing method, thereby ensuring higher quality for the end consumer while also simplifying quality assurance in the meat processing industry. We also envisage the CampyTube principle as a versatile and extremely helpful test format for various applications in infectious disease diagnostics.

## 2. Materials and Methods

The majority of the chemicals for the buffers (PBS and Tris-HCl components, potassium monophosphate, potassium chloride, polyethylene glycol (PEG), bovine serum albumin (BSA), casein, Tween 20, and trehalose), as well as streptavidin, were purchased from Carl Roth, Karlsruhe, Germany. Moreover, 1-ethyl-3-(3-dimethylaminopropyl)carbodiimid-hydrochlorid (EDC) and N-hydroxysulfosuccinimide (sulfo-NHS) were bought from ThermoFisher, Waltham, MA, USA, and hydroxylamine from Sigma Aldrich, Schnelldorf, Germany.

The 150 nm gold nanoshells were obtained from NanoComposix, San Diego, CA, USA (product number GSXR150). The nitrocellulose membrane CN95 was ordered from Sartorius, Göttingen, Germany; the conjugate and absorbent pads from Ahlstrom, Helsinki, Finland; and the backing cards DCN 0.010”from DCN, Carlsbad, CA, USA. The manufactures of the antibodies and their specific product numbers are indicated in the protocols below. The two-component silicone for the manufacturing of the tube inserts was purchased from S. u. K. Hock, Regen, Germany.

The reagents for DNA extraction and qPCR were produced by Hygiena Diagnostics, Potsdam, Germany, as also referred to in the specific procedure steps.

Deoxyribonucleotides (dNTPs), magnesium sulfate, and Bst 2.0 polymerase for the loop-mediated isothermal amplification (LAMP) stemmed from New England Biolabs, Frankfurt am Main, Germany. The primers were ordered from Eurofins, Ebersberg, Germany.

The integration of the CampyTube test was performed in 0.5 mL screw cap tubes from Bio-Rad, Feldkirchen, Germany.

The chicken skin samples for the test evaluation were kindly provided by Plukon Storkow GmbH, Storkow, Germany.

The analysis of the sensitivity and specificity was performed in GraphPad Prism for Windows, Version 6.07 (GraphPad Software, Boston, MA, USA).

### 2.1. Manufacturing of Mini LFT

The conjugate for the mini LFT was produced via the conjugation of 35 µg of mouse anti-FITC antibodies (Abcam, Cambridge, UK, product number ab10257) to 1 mL of gold nanoshells via EDC-NHS cross-linking following the EDC product instructions [15]. Ten µL of hydroxylamine was added and incubated for 10 min to quench the reaction. The final conjugates were stored at 4 °C in conjugate diluent (0.5× PBS, 0.5% BSA, 0.5% casein, 1% Tween 20, 0.05% sodium azide, and pH 8.0).

For the manufacturing of the 25 mm mini LFTs, nitrocellulose membrane CN95 was mounted on 25 mm-wide backing cards DCN 0.010”. Conjugate and absorbent pads were cut to an 8 mm width. Conjugates were dispensed at a 25 µL/cm ratio using the Automated Lateral Flow Reagent Dispenser (ALFRD, Claremont BioSolutions, Upland, CA, USA). Streptavidin and goat anti-mouse IgG (Sigma Aldrich, product number M8642, Germany) were striped as test and control lines, respectively, using the S3 FlexArrayer (Scienion, Berlin, Germany) at 0.5 µL/cm. The conjugate and absorbent pads were mounted on the backing card with the membrane.

### 2.2. Manufacturing of the Tube Insert: Mini LFT Holder

The tube inserts, featuring a fitted slit for integrating the mini LFT, were designed to fit tightly into the lower portion of a standard 0.5 mL vial without coming into contact with the liquid reaction mix at the bottom of the tube. They were fabricated using a 3D-printed mold treated with a mold release agent (Figure S1). A two-component silicone was mixed in a 1:1 ratio, poured into the mold, and removed after curing for 30–60 min at room temperature.

### 2.3. Enumeration of Campylobacter in the Samples via Microbiological Culture

The microbiological detection of *Campylobacter* was based on the guidelines from EN ISO 10272-1 [16]. Chicken skin samples were trimmed; ~10 g of the sample was rinsed with 9 weight parts of buffered peptone water, stomached for 1 min, and settled for 5 min. Tenfold serial dilutions were made in peptone water; 0.1 mL of the dilutions was plated onto a 9 cm modified Charcoal-Cefoperazone-Deoxycholate (mCCD) agar plate. Characteristic colonies were counted after ~48 h of incubation in microaerophilic conditions at 41.5 °C.

### 2.4. Sample Preparation for LAMP and PCR

Sample preparation was based on rapid DNA extraction with the StarPrep^®^ Three Kit (Hygiena Diagnostics), with additional pre-treatment steps with Reagent D (Hygiena Diagnostics) to detect only viable *Campylobacter* spp. This light-sensitive reagent penetrates dead cell membranes and binds to DNA upon light exposure, blocking the PCR amplification of DNA from dead cells [17].

Then, 800 µL of peptone-rinsed sample, as prepared for microbiological culture, was mixed with 100 µL of Reagent D and incubated in the dark for 10 min, followed by 5 min light exposure in the foodproof D-Light instrument (Hygiena Diagnostics). The sample was centrifuged for 10 min at 5400× *g*, and the supernatant was discarded. The pellet was resuspended in 200 µL of StarPrep^®^ Three Lysis buffer containing 1.25% *v*/*v* BAX proteinase (Hygiena Diagnostics). The sample was incubated for 10 min at 37 °C and subsequently inactivated at 100 °C for 10 min. After cooling to room temperature, the sample was spun down for 5 min at 5400× *g*. Twenty-five µL of the supernatant was used for qPCR or the CampyTube test.

**Figure 1:**
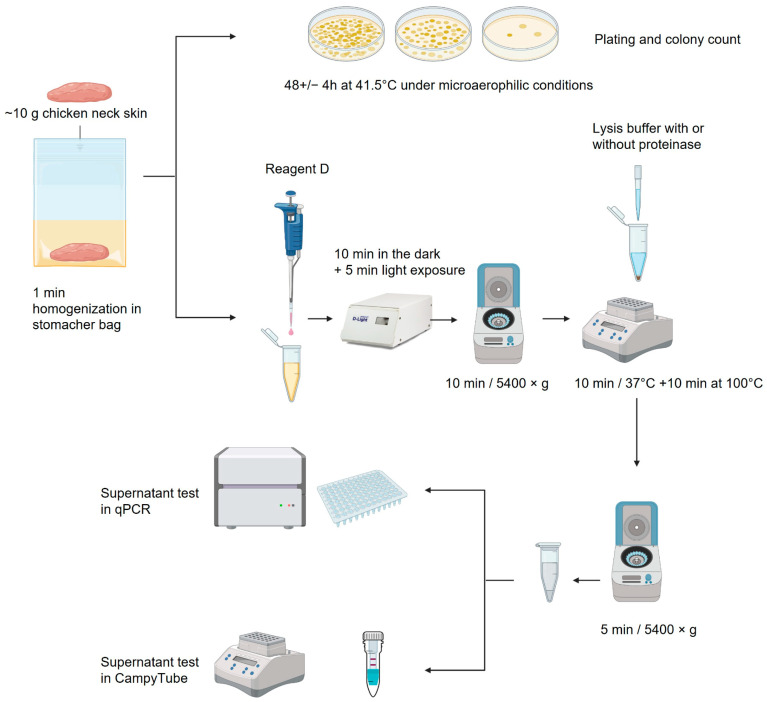
Schematic overview of the sample processing: description in text [18].

Alternatively, a treatment without the addition of proteinase was also investigated in paired samples. The treatment steps remained unchanged, except for the omission of the enzyme. A schematic overview of the sample preparation is shown in Figure 1.

### 2.5. LAMP Reaction with Lateral Flow Detection

The LAMP assay targeted a region of the thermotolerant *Campylobacter* spp. (Accession number Y19244). The primer sequences published by Romero et al. were used in this study [19].

The reaction mix contained 20 mM of Tris-HCl pH 8.8, 50 mM of KCl, 8 mM of magnesium sulfate, 1.4 mM of each deoxynucleotide (dNTPs), 0.25 mg/mL of BSA, 6% (*w*/*v*) trehalose, 0.2 µM each of the primers B3 and F3, 1.6 µM each of the primers BIP and FIP, 0.4 µM each of the labeled loop primers 5′-Biotin-LF and 5′-FAM-LB, and 320 U/mL of Bst 2.0 polymerase. The primer sequences were designed and published by Romero et al. [19] and are listed in Table S1. The reagents for the LAMP assay were freeze-dried in 0.5 mL screw cap vials in portions for 25 µL of reaction volume in an Epsilon 2–6 Freeze Dryer (Christ, Osterode am Harz, Germany).

For the initial characterization of the test performance, lateral flow detection was performed in a non-integrated format, either using the in-house produced mini LFT or commercially available LFT Amodia DetectLine Basic (Amodia, Braunschweig, Germany). The detection was performed in a separate closed area to prevent carry-over contamination with the products of DNA amplification. The mini LFTs (25 × 3 mm) were placed in the reaction vial with the 25 µL LAMP product; the bands were visible after ~1 min. The commercial 60 × 4 mm LFT required a larger detection volume, so 100 µL of chromatographic buffer 01 (Amodia, Germany) was added to the reaction vial with the 25 µL of LAMP product. The LFT was placed in this sample and the result was registered after ~10 min, when the bands had distinctly formed and the background had cleared.

### 2.6. CampyTube Assembly and Application

Twenty-five µL of the sample was pipetted into the reaction vial with the freeze-dried LAMP mix. For the determination of the limit of detection, 24 µL of water and 1 µL of purified *Campylobacter jejuni* DNA or 25 µL of water as a negative control were used. A mini LFT was placed into the slit of the vial insert, and this complex was embedded into the vial to fit tightly. The vial was closed with the screw cap and incubated in a heating block HB-2 (Wealtec, Taiwan) for 40 min at 65 °C.

After incubation, the vials were removed from the heating block, flipped over, and lightly snapped with the finger to force the liquid flow towards the mini LFT. The vials were left in the upside-down position for ~1 min until the test and/or control bands became visible. The results were interpreted qualitatively by the naked eye; pictures of the LFT results were taken for documentation.

### 2.7. qPCR

The qPCR was performed using a commercial kit prototype (Hygiena Diagnostics). The kit targets sequences specific to the entire group of thermotolerant *Campylobacter* species (FAM), as well as the individual species *C. jejuni* (HEX), *C. coli* (ROX), and *C. lari* (melt curve, FAM). The kit additionally contains an internal control amplification (Cy5) to monitor PCR inhibition. The qPCR mix in the kit is provided in lyophilized form for 25 µL of reaction volume in individual wells on a PCR plate. Furthermore, 25 µL of the sample was added to the reaction well. PCR was performed in a LightCycler 480 II (Roche, Mannheim, Germany). The reaction started with 4 min pre-incubation at 37 °C for uracil-DNA Glycosylase (UNG)-mediated decontamination, followed by initial denaturation for 5 min at 95 °C and 50 cycles of two-step amplification (5 s at 95° + 60 s at 60 °C). Fluorescence detection was performed at the end of the annealing step. A melting curve acquisition from 40 to 65 °C was added after the run to specifically detect the presence of *C. lari*. A digital PCR-quantified DNA standard containing the thermotolerant *Campylobacter* target sequence in four decadic dilutions from 10^1^ to 10^4^ genomic copies per reaction was included in every run. FAM-channel data from these standards were used to construct a standard curve for the quantification of the total thermotolerant *Campylobacter* in field samples. Quantitative interpretation of the results was performed for the samples ≥ 5 copies per reaction, which had been verified as a suitable limit of quantification with an acceptable E_LQ_ ≤ 0.25.

## 3. Results

### 3.1. Development of Mini LFT

The development of the closed-tube mini LFT was driven by three requirements for the fully integrated format: (1) its dimensions must allow for fitting into a standard 0.5 mL reaction vial, (2) its activation must require ≤ 50 µL liquid, and (3) its sensitivity must not be compromised by the dimensions. The developed test has the dimensions of 25 mm × 3 mm, which was enabled not only by shrinking the sizes of the LFT elements, but also by sacrificing the sample pad, i.e., the conjugate pad in the test serves for the sample uptake. Twenty-five µL of liquid, the entire volume of the LAMP reaction, was sufficient for the effective detection of the labeled LAMP product: distinct test (T) and control (C) bands were visible, and the background was cleared without the addition of any chromatographic buffer.

**Figure 2:**
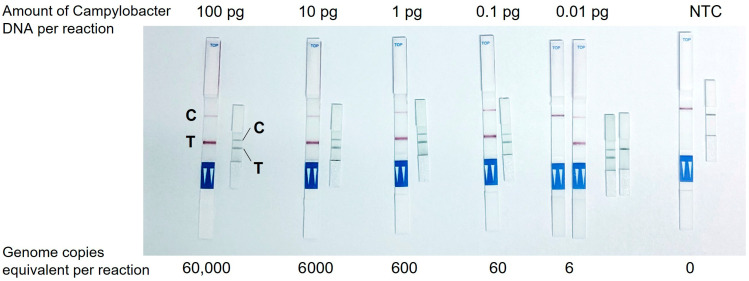
Detection of *Campylobacter* DNA amplified in LAMP with a commercial LFT and newly developed mini LFT. C, control band, T, test band.

The efficacy of the mini LFT was investigated in comparison with a conventional commercially available test strip that had dimensions of 60 mm × 4 mm and included all standard components of an LFT, i.e., a sample pad, a conjugate pad, a backed nitrocellulose membrane, and a wicking pad. The use of the commercial LFT required the addition of the chromatographic buffer to the LAMP product. The mini LFT demonstrated a similar performance to the commercial LFT: as little as 0.1 pg (60 genome copies) of *Campylobacter* DNA amplified in LAMP was stably detected, resulting in bright T-bands on both tests, while lower serial DNA dilution already lacked detection reproducibility, also with both tests (exemplary demonstration in Figure 2). It was noteworthy that the time-to-readout (i.e., the time from the LFT contact with the LAMP product to the complete clearing of the GNP background on the membrane and optimal visibility of the bands) was significantly shorter on the mini LFT, namely, ~1 min vs. ~10 min on the commercial test strip.

### 3.2. Integration of the Test Components and Processes in Fully Closed Campytube Format

**Figure 3:**
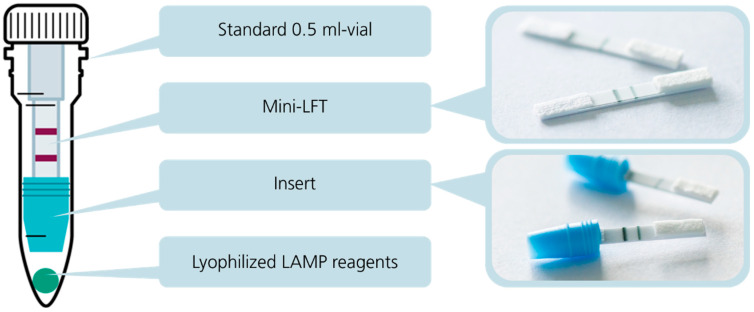
Structure of the CampyTube: Schematic assembly and its separate elements, a mini LFT and a mini LFT placed into a vial insert.

CampyTube is a fully closed test format for the molecular amplification and detection of *Campylobacter* nucleic acids that consists of three functional elements: a mini LFT, a vial insert (a mini LFT holder), and lyophilized LAMP reagents, integrated in a 0.5 mL reaction vial (Figure 3). The vial is a standard laboratory disposable item that enables the use of the CampyTube with common equipment and heating blocks and also obviates the need for any customized design and manufacturing of the test housing.

**Figure 4:**
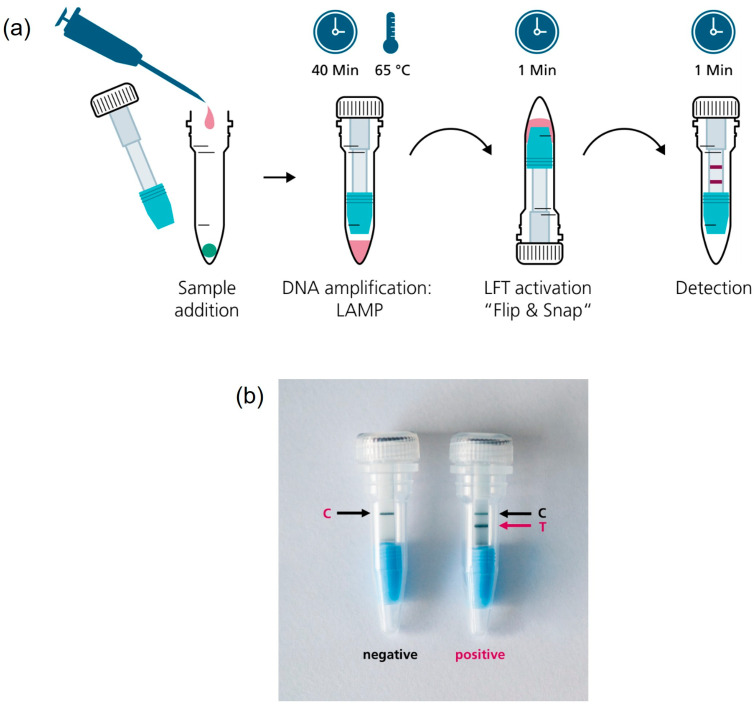
(**a**) CampyTube workflow; (**b**) Detection of the test result on the integrated mini LFT. T, test band, C, control band.

The vial insert was designed to fulfill several key functions in the CampyTube. First, it serves for the optimal positioning of the mini LFT in the vial, providing the necessary distance between the liquid reaction and the test strip. Second, it enables using a low reaction volume (here: 25 µL) without its evaporation during the heating step (65 °C for 40 min) via the tight sealing of the bottom part of the vial. Thus, although the vial itself is relatively large and could potentially lead to vapor condensation and the loss of the effective LAMP volume, the reaction runs within a small segregated compartment that eliminates these problems. We did not notice any significant condensation of the liquid during the tests, and no pre-term wetting of the mini LFT occurred. Third, the bottom part of the insert has a funnel shape that efficiently directs all the sample volume towards the mini LFT. For the targeted LFT activation after nucleic acid amplification, the vial must be flipped over and lightly snapped to let the liquid sample run into the insert towards the mini LFT (Figure 4). The “Flip & snap” mechanism activates the LFT only in the upside-down vial position and ensures that no spontaneous activation takes place during the handling or eventual dropping of the vials. This handling security is naturally maintained via liquid tension; only the intentional snapping of the vial (not dropping, shaking, or flipping alone) breaks the tension and allows the liquid to flow directly toward the LFT. The shape of the insert prevents any by-pass of the liquid and ensures reproducible and effective detection with a low sample volume.

**Figure 5:**
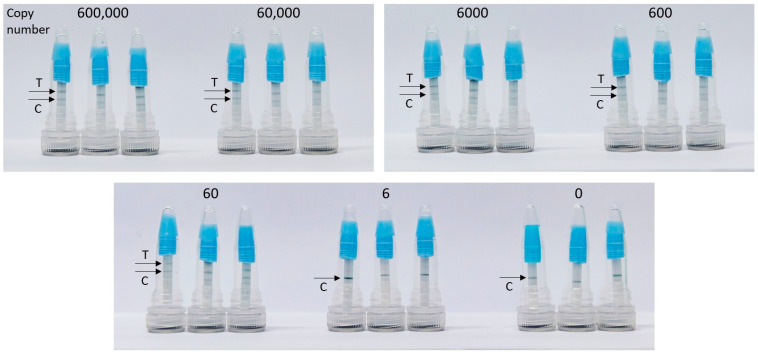
Detection of *C. jejuni* DNA in the integrated CampyTube. T, test band, C, control band.

The limit of *Campylobacter* DNA detection in the integrated CampyTube format remained the same as in the bench protocol (see Figure 2): sixty genome copies were successfully detectable (Figure 5).

### 3.3. Detection of Campylobacter in Chicken Skin Samples with CampyTube and qPCR

Sixty rinsed chicken skin samples prepared according to the specifications of Commission Regulation (EU) 2017/1495 were tested for the presence of *Campylobacter* in CampyTube using qPCR as the reference method. Two sample preparation protocols were compared for their impact on the detection efficiency: (1) without proteinase treatment and (2) with proteinase treatment. The first, simplified version of the protocol with omitted proteinase treatment was evaluated as a minimalistic time-saving procedure. However, its performance proved to be insufficient: only 34 out of 60 samples were detected as positive, resulting in a sensitivity of 61.8% and specificity of 88.5%.

In contrast, when proteinase treatment was included in the sample preparation, the detection efficiency improved significantly. Among the 45 out of the 60 samples that tested positive via qPCR (containing 1.4 × 10^2^ to 2.5 × 10^6^ genome copies/g meat), the sensitivity increased to 71.1% while maintaining 100% specificity. The sensitivity reached 100% for all the samples (17/17) containing > 1000 CFU/g, confirmed microbiologically (a hygienically relevant pathogen load in poultry meat proposed by the Commission Regulation (EU) 2017/1495) [10]. In the same subgroup of samples without proteinase, the sensitivity remained low and reached only 76.5%, thus demonstrating the clear advantage of using proteinase treatment for improved test performance.

**Figure 6:**
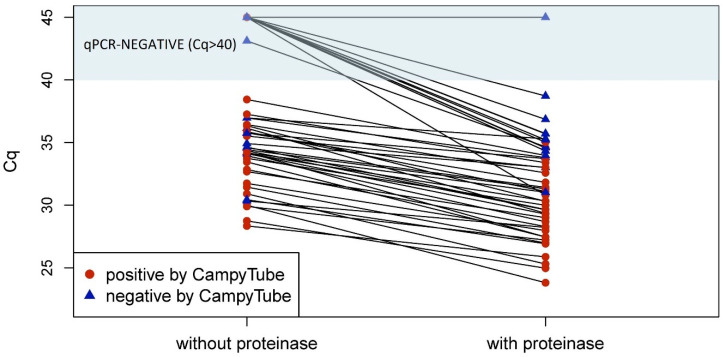
qPCR and CampyTube results in paired samples with and without proteinase treatment. A cut-off at Cq = 40 was applied to qPCR results.

The positive impact of the proteinase treatment on the detectability of *Campylobacter* in individual rinsed meat samples is visualized in the plot in Figure 6. It demonstrates that in all cases, the efficiency of qPCR detection was always higher with proteinase (lower Cq values in paired samples except for one sample that remained negative). Moreover, without the proteinase treatment, some samples tested negative in PCR and/or CampyTube despite a high pathogen load in the specimen. In most cases, a clearly positive result was obtained for these samples after the proteinase treatment.

## 4. Discussion

According to the Agricultural Outlook 2023–2032, the global meat supply will continue to expand over the next 10 years, reaching 382 million tons by 2032 [20,21]. In the upper middle-income countries, the production growth will increase by +14%. Poultry meat will account for half of this expansion in the next decade. The growing world population combined with the improvements in animal breeding and meat production technologies are the main drivers of this growth. [21]. The high density of poultry production bears high risks of increasing the prevalence of associated infections, such as Campylobacteriosis. Biosecurity measures and hygiene control at all stages of the meat production chain are therefore essential for the prevention of public health risks.

We have developed a simple and cost-effective format for the molecular detection of pathogens at the point of need. CampyTube is a first-of-its-kind seamless integration format of an LFT with molecular detection in a completely closed single vial. This is realized with two innovative components: a specially designed vial insert and a miniaturized LFT. The insert and the mini LFT enable integrated DNA amplification and its immediate subsequent detection in only 25 µL of liquid, without any additional buffer or further manual steps. All test components, from the dry amplification reagents to the mini LFT, are incorporated in a single vial, which is closed after the addition of liquid sample and never has to be opened again. This ensures the absolute prevention of carry-over contamination and makes the system very safe and simple to use in point-of-care settings. The readout of the results is performed by the naked eye; the test and control bands are visible through the transparent wall of the vial.

CampyTube was developed to speed up hygiene control in poultry production facilities. Conventional methods for *Campylobacter* detection include the microbiological culture of the pooled meat samples according to EN ISO 10272-2 [22]. This procedure is labor intensive and takes 48–72 h, while the processing of meat, its packaging, and delivery to consumers is already executed during this time. Hence, microbiological methods cannot enable a fast response to an eventual significant contamination in meat production facilities, so rapid detection methods are sought. The latest amendment to the EN ISO 10272-2 (A1:2023) refers to the use of molecular methods, particularly PCR, for the detection of *Campylobacter* spp. in the food chain. While being a faster method (2–3 h), PCR requires specially equipped molecular diagnostic laboratory and skilled personnel at the point of need; otherwise, the time-to-result is extended by the time of the sample transportation to an external laboratory.

Multiple reports address the development of LFT-based assays for the detection of foodborne bacteria in various sample matrices, from feces to milk products, including fresh, frozen, and processed meat. The most recent reviews describe the broad span of the achievable limits of detection, from 1 CFU/mL to ~10^10^ CFU/mL [23,24,25]. The limits of detection <100 CFU/mL mostly required the combination of a lateral flow assay either with signal enhancement, like fluorescent nanoparticles, nanozymes, quantum dots, magnetically, or electrochemically active labels, among others, and were coupled with an instrumental readout, and/or were combined with nucleic acid amplification like our method, yet lacked a fully integrated format. Ji et al. underline that such a kind of detection of bacterial nucleic acids on LFT “greatly improves the detection sensitivity and specificity, but the detection process is more complicated and susceptible to false positives” [23]. The latter challenge has motivated us in the development of the fully closed system for the detection of amplified nucleic acids in an instrument-free manner.

CampyTube can be used with a simple heating block as the only required instrument (the use of a centrifuge during sample preparation is considered to be optional for CampyTube; its use here was primarily driven by the needs of qPCR). The entire duration of the detection takes ~60–75 min; of this, 20–35 min is sample preparation (depending on the optional use of Reagent D), 40 min amplification, and ~1 min readout of the result. Sample preparation turned out to be a significant factor for the effective detection of *Campylobacter* both via qPCR and CampyTube. First, the sensitivity of *Campylobacter* detection in rinsed meat samples after their treatment with proteinase was higher than without the enzyme (Table 1). Beyond this fact, the absence of a correlation between the pathogen load and a negative result in PCR and/or CampyTube without the proteinase treatment suggests that the negative results are caused by the inhibition of DNA amplification in individual specimens. The successful neutralization of the inhibitors via the proteinase treatment allows them to assume their protein nature—either a total amount of protein or most likely nucleases may play a crucial role in the inhibition of DNA amplification. No detailed investigation of these aspects was performed in our study, yet we assume that the assay sensitivity may have been negatively affected by some false-negative results in CampyTube due to the inhibition of amplification. Besides the already mentioned types of inhibitors, other substances like fat content or blood traces may play a significant role. The eventual inhibition of the reaction in some samples implies the need for the introduction of an internal amplification control in the CampyTube. This feature is easily implementable via the addition of a synthetic target and primers to the LAMP mix and the introduction of an additional internal control line on the test strip. In order to enable good differentiation by the naked eye on the mini LFT, a different color or shape of this line may be used. We aim to add this feature to upcoming versions of the test format.

Worth mentioning is the option of speeding up the amplification time in CampyTube in the outlook. Novel bioengineered polymerases have become available recently and should enable a potential reduction in the amplification time to ~20 min, according to the information from the enzyme manufacturers (to be evaluated).

Further adjustment of the test can be implemented for the complete alignment of its sensitivity, with cut-off values specified in regulatory standards. The European Food Safety Authority (EFSA) proposes quantitative hygiene criteria that would reduce the public health risks associated with *Campylobacter*. According to EU legislation [10], adhering to a *Campylobacter* limit of 1000 CFU/g in chicken carcasses could reduce the public health risks associated with poultry consumption by over 50%. In our small-scale evaluation with a limited number of samples, CampyTube has shown 100% sensitivity for samples with >1000 CFU/g of *Campylobacter*. In order to use CampyTube for testing the compliance to this hygienic limit, lower concentrations should not be detectable by the test. While in the current study this cut-off was not integrated yet for the sake of a comprehensive test evaluation, it is technically possible to adjust the sensitivity to the desired limit of detection (LOD). For instance, this could be approached via a reduction in the duration of DNA amplification (see Supplementary Information, Table S2) or an adjustment of the LAMP primer concentration.

In our study, qPCR could detect more *Campylobacter*-positive samples than CampyTube, and enabled the precise quantification of viable *Campylobacter* (genome copies) in the samples, but it lacked the technical simplicity necessary for application at the point and time of need. Some available alternatives to PCR among the *Campylobacter* tests are also based on isothermal amplification, and therefore require less complex instrumentation in comparison to real-time PCR devices. Lin et al. presented a prototype of a system for smartphone-based *Campylobacter* detection in LAMP [26]. However, it requires a specialized device for the integration of heating and readout. Moreover, the use of a smartphone for the quality control, certification, and validation of a diagnostic test remains problematic due to the varying technical characteristics of different smartphone models. The Neogen^®^ Molecular Detection Assay (Neogen Corporation, Lansing, MI, USA) is a commercial LAMP assay that can be run in a compact instrument, yet it requires the pre-enrichment of bacteria in a nutritional broth [27], as other proposed LAMP methods do [28,29,30]. At the same time, Romero and Cook (2018) have demonstrated that the detection of *Campylobacter* in chicken carcasses via LAMP is also possible without enrichment, which aligns well with our results [31]. The authors also proposed a convenient sampling method by swabbing meat and reached a detection limit of 800 CFU/swab. While it is not possible to assess this LOD in relation to the recommended limit of 1000 CFU/g due to the unstandardized size of the swabbed chicken carcasses, this method can be an attractive sampling technique to be combined with CampyTube, since it obviates more laborious rinsing and very likely reduces the proportion of the inhibitors in the sample. A 5 min heating at 85 °C was sufficient to inactivate the presumably present inhibitors in this study. A quantitative characterization of the combination of swab sampling and CampyTube could potentially enable even more rapid point-of-care testing. Feng et al. have also confirmed that cloth sampling yields identical results to the rinse method in the detection of Salmonella in meat samples, thus highlighting more convenient alternative sampling strategies [32].

Yet none of the isothermal amplification methods described above enabled a truly integrated simple point-of-care format: The reported developments are rather based on typical laboratory procedures with the manual setup of the reactions. The development of CampyTube was driven by the motivation to provide the simplest and most convenient possible molecular test format for non-proficient users or settings with limited technical and personnel resources. To our knowledge, CampyTube is the first-of-its-kind system that seamlessly combines molecular nucleic acid amplification and lateral flow detection in one standard laboratory vial in a single-step, fully integrated test. While lateral flow detection offers straightforward instrument-free detection by the naked eye, its application for DNA amplicons bears a high risk of carry-over contamination between the tests. Typical LFTs require the addition of a detection buffer or operate with a large (≥100 µL) total sample volume. The only reported system aiming to address the challenge of sealed lateral flow detection of amplified products is a UStar Nucleic Acid Probes (fragments) Detection Device (Ustar Biotechnologies, Hangzhou, China) [33]. However, it comprises a multi-component plastic cassette that needs to be pre-loaded with the vial of the detection buffer and serves only for the detection of the products amplified externally elsewhere, while being rather bulky and thus also expensive, and producing a considerable amount of plastic waste. Since CampyTube uses a common amplification vial as housing for closed lateral flow detection, our system is a sustainable option for a variety of point-of-care applications. The amount of plastic in such a vial is only 15% of the amount used in typical LFT cassettes (~0.95 g vs. ~6.35 g, compared to an exemplary test cassette (DCN, USA)). Remarkably, the production of the mini LFT requires ~3× less nitrocellulose than an average LFT, making CampyTube a cheaper and more sustainable rapid test.

Another product that combines integrated DNA amplification and lateral flow detection is Multitest COVID-19 (Selfdiagnostics, Leipzig, Germany) [34]. Both processes occur within a sealed microfluidic chip. The activation of the LFT in the Multitest requires the addition of the detection buffer, which is preloaded in a blister compartment on the cartridge, and its release is triggered by applying pressure to the blister. However, the cartridge is relatively large (approximately hand-sized) and contains disposable heating elements, as the amplification process operates at ~65 °C. Due to its size and design, regular use in point-of-care (PoC) applications would not be sustainable. Additionally, the complexity of the system results in high production costs, thus making the device unsuitable for high-throughput areas like hygiene control. We believe that the users of the CampyTube format can especially benefit from its integration into a standard disposable 0.5 mL lab vial due to its cost-saving manufacturing and compatibility with any standard heating blocks or racks. This format is especially favorable for application areas with high throughput or low resources. In the current study, we demonstrated a functional prototype of the system where the insert was produced manually; Figure S2 provides an idea of a single-piece insert merged with the cap. Upon need, the system allows for integration in various reaction vessels and volumes with minimal design adjustments.

Among the few non-integrated and thus cheap variants of LAMP-based tests amenable for detection by the naked eye are colorimetric LAMP tests [35,36]. Commercially available colorimetric LAMP assays utilize pH-sensitive dyes to visually indicate DNA amplification through color changes. Yet the weak buffering capacity of pH-based colorimetric LAMP master mixes can limit sample compatibility, as highly buffered or acidic samples may impact the color change, leading to unspecific results [37]. Visual interpretation of the indistinct color change by the operator can be incorrect and depends on the human factor. Furthermore, colored samples like blood can mask the color change completely. CampyTube enables working with such samples: as long as the LAMP itself is not inhibited, detection remains unaffected and unbiased. Our ongoing tests (unpublished) have demonstrated the direct compatibility of CampyTube with up to 8% *v*/*v* blood in LAMP reactions. Thus, CampyTube offers advantages over the available simple colorimetric LAMP tests, especially considering the potential of CampyTube for multiplexing (detection via multiple bands on the LFT), which is not possible in colorimetric reactions.

## 5. Conclusions

CampyTube enables the seamless integration of isothermal DNA amplification with instrument-free qualitative detection via a mini LFT placed inside a conventional 0.5 mL reaction vial. The main advantage of this system is its secure and simple use without the risk of carry-over contamination. The compact format is material-saving and convenient for logistics and application. The system is versatile and can be adapted for other pathogens. The novel test has demonstrated 100% sensitivity in chicken skin samples, exceeding the regulatory limit of 1000 CFU/g, with 71.1% overall sensitivity and 100% specificity. The current limit of detection is 60 genome copies/reaction. In the outlook, it would be of importance to integrate an internal amplification control into the test, as well as to evaluate the system with larger sample quantities and other sample matrices. We believe that this test format can enable safe and reliable molecular diagnostics in many point-of-care application areas.

## 6. Patents

German patent DE102024103902 [38]; international patent applications are filed.

## Figures and Tables

**Table 1:** Sensitivity and specificity of CampyTube test in rinsed chicken meat samples. PPV, Positive Predictive Value, NPV, Negative Predictive Value, and CI, confidence interval.

Without Proteinase Treatment		
Number of Samples	Positive via qPCR	Negative via qPCR
Positive via CampyTube	21 (of those, 13 with >1000 CFU/g)	3
Negative via CampyTube	13	23
Total:	34	26
	60	
Diagnostic performance	Value (95% CI)	
Sensitivity	61.76% (43.6–77.8%)	
Sensitivity in samples with >1000 CFU/g	76.5% (50.1–93.2%)	
Specificity	88.5% (69.9–97.6%)	
PPV	87.5% (67.6–97.3%)	
NPV	63.89% (46.2–79.2%)	
With Proteinase Treatment		
Number of samples	Positive via qPCR	Negative via qPCR
Positive via CampyTube	32 (of those, 17 with >1000 CFU/g)	0
Negative via CampyTube	13	15
Total:	45	15
	60	
Diagnostic performance	Value (95% CI)	
Sensitivity	71.1% (55.7–83.6%)	
Sensitivity in samples with >1000 CFU/g	100% (85–100%)	
Specificity	100% (78.2–100%)	
PPV	100% (89.11–100%)	
NPV	53.6% (33.9–72.5%)	

## Data Availability

The original contributions presented in this study are included in the article and Supplementary Material. Further inquiries can be directed to the corresponding author.

## Supplementary Materials

The following supporting information can be downloaded at: https://www.mdpi.com/article/10.3390/bios15080497/s1. Figure S1: Design of the tube insert and the negative mold for its manufacturing; Figure S2: An option of the insert design and its integration as a single piece merged with the vial cap; Table S1: Primer sequences used in this work; Table S2: LAMP detection time for different concentrations of *Campylobacter* DNA.

## Abbreviations

The following abbreviations are used in this manuscript:

CFU	Colony-forming unit
LAMP	Loop-mediated isothermal amplification
LFT	Lateral flow test
LOD	Limit of detection
