# Supplementary material for: CampyTube: Seamless Integration of a Molecular Test and Lateral Flow Detection of Campylobacter in a Single Vial
Source: Biosensors (Basel). 2025 Aug 1;15(8):497. doi: 10.3390/bios15080497 (PMC12384175; doi:10.3390/bios15080497)
Supplement: Supplementary file 1 [file biosensors-15-00497-s001.zip › biosensors-3735334-supplementary.pdf]

Supplementary information to the manuscript "**CampyTube: A seamless integration of a molecular test and lateral flow detection of Campylobacter in a single vial**"

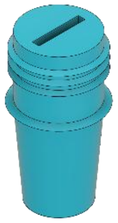

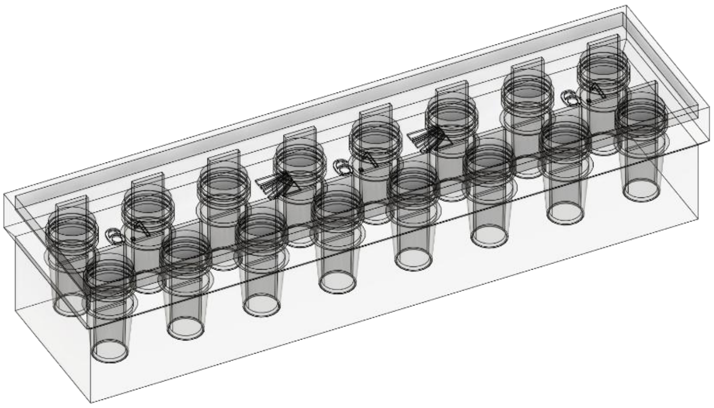

Figure S1. Design of the tube insert (left) and the negative mold for its manufacturing (right).

Table S1. Primer sequences used in this work (M. R. Romero et al., 2016).

Primer type	Primer sequence 5'-3'
F3	CTGCTTAACACAAGTTGAGTAGG
B3	TTCCTTAGGTACCGTCAGAA
FIP	GGACCGTGTCTCAGTTCCAGTGTGACGGATGAGACTATATAGTATCAGCTAG
BIP	CGGGAGGCAGCAGTAGGGAATATTGCTAAGAAAAGGAGTTTACGCTCCG
LF	GTTAAGCGTCATAGCCTTGGTAA
LB	GCGTGGAGGATGACACTT

Table S2. LAMP detection time for different concentrations of Campylobacter DNA.

Campylobacter DNA, pg per reaction	positive/tested	Time-to-positivity
10	3/3	27.5 min
1	3/3	30 min
0.1	3/3	33 min
0.01	1/3	~35.5 min

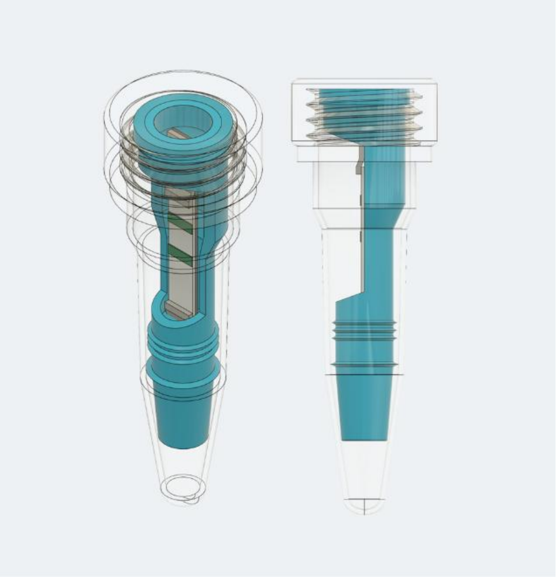

Figure S2. An option of the insert design and its integration as a single piece merged with the vial cap.
